# Editorial Comment to Laparoscopic partial nephrectomy for the horseshoe kidney with indocyanine green fluorescence guidance under the modified supine position

**DOI:** 10.1002/iju5.12456

**Published:** 2022-04-26

**Authors:** Toru Kanno

**Affiliations:** ^1^ Department of Urology Ijinkai Takeda General Hospital Kyoto Japan

Intraoperative indocyanine green (ICG) near‐infrared fluorescence guidance is a type of optical imaging technology currently available to facilitate a better understanding of surgical landmarks. Therefore, ICG has been utilized intraoperatively during laparoscopic or robotic urologic procedures to help identify relevant anatomy and assess perfusion.^1^ Indeed, the urologic oncologic and non‐oncologic applications of ICG are vast, such as partial nephrectomy, lymph node dissection, and the reconstruction of urinary systems.^1^ Particularly, ICG may be extremely useful for unusual cases because the information from ICG findings can improve our understanding of the unique anatomy including blood supply. In this respect, we have recently reported the application of ICG for the laparoscopic lower‐pole heminephrectomy for duplex kidney^2^ and partial cystectomy and pelvic lymphadenectomy for urachal carcinoma.^3^

In this article, Imai et al described the useful application of ICG for the horseshoe kidney during laparoscopic partial nephrectomy.^4^ For cases unfamiliar and anatomically complex and difficult for most urologic surgeons, the authors have performed excellent surgery, which deserves praise. Basically, surgical management of renal cell carcinoma in horseshoe kidney can be highly challenging due to the limited kidney mobilization and access to the renal hilum, in addition to the abnormal and highly variable vessels.^5^ ICG technique can be of particular use in renal cancers of horseshoe kidney because these are the tumors where intraoperative visualization of aberrant vessels can be extremely advantageous to assess whether arterial clamping results in sufficient ischemia prior to the resection of the tumor.

In addition, the authors reported that retroperitoneal laparoscopic partial nephrectomy was successfully performed in the modified supine position and modified port placement, which provided an excellent operative field. The authors might choose a retroperitoneal approach with the modified supine position because of the large experience with radical nephroureterectomy in this position for upper tract urothelial tumors,^3^ but urologists are usually unfamiliar with the supine retroperitoneal approach. However, this report tells us that we should master this approach since it is very useful in some unique situations.

## Conflict of interest

The author does not have any conflicts of interest to declare.
